# Tick infestation on the lower eyelid: a case report

**DOI:** 10.1186/1757-1626-2-9073

**Published:** 2009-11-23

**Authors:** Vasilis Liolios, Craig Goldsmith

**Affiliations:** 1 Hull Royal Infirmary NHS Trust, Anlaby Road, Hull, HU3 2JZ, UK; 2 James Paget University NHS Foundation Trust, Gorleston, Norfolk, NR31 6LA, UK

## Abstract

**Background:**

The tick infestation of the lower lid is a quite rare condition.

**Case Presentation:**

We report a case of a 40 years old caucasian female who presented with the above condition after camping in the Norfolk area, UK.

**Conclusion:**

Tick bite can be responsible for many diseases the most common being Lyme disease which can affect the eyes in several ways. It is still debatable whether or not prophylactic treatment is needed after tick bite.

## Background

To report the rare case of tick infestation on the lower eyelid.

## Case Presentation

**Figure 1:**
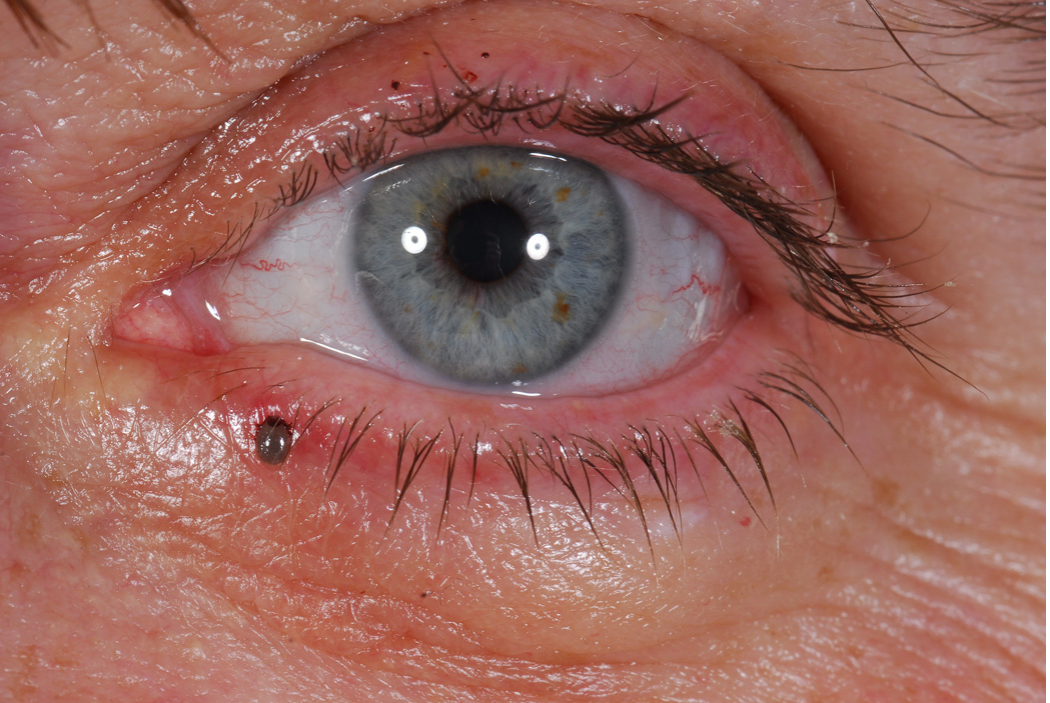
**lower lid tick infestation picture**.

**Figure 2:**
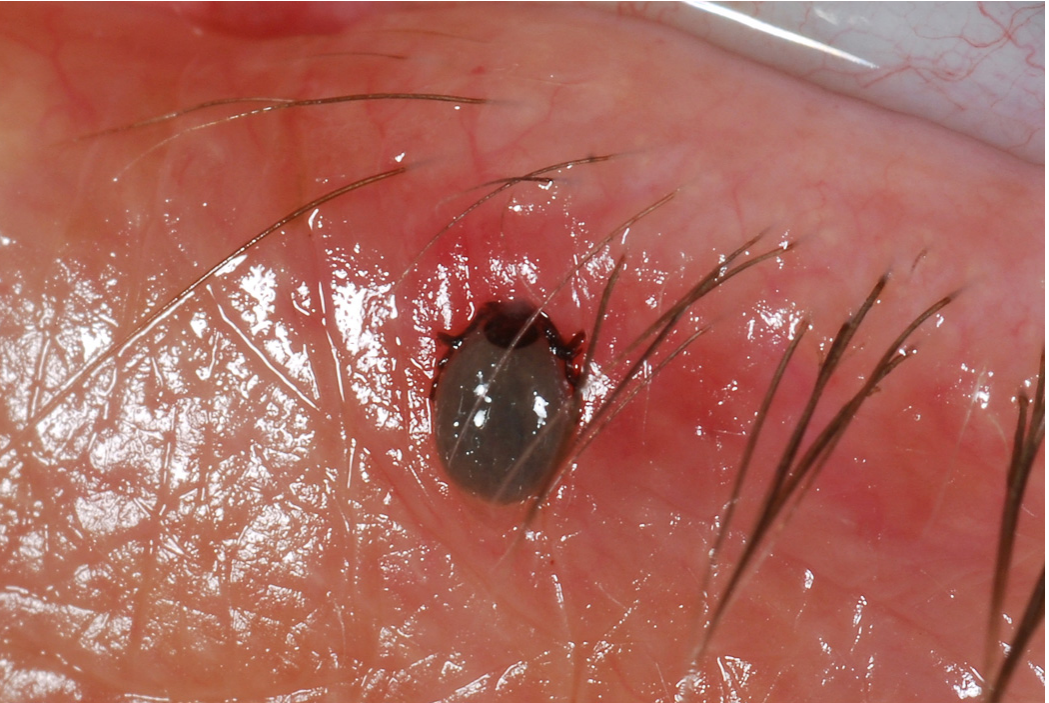
**lower lid tick infestation picture**.

A 40 years old female Caucasian presented in the eye casualty with a tick on her left lower eyelid after a weekend camping in the Norfolk area. Fig 1, 2, 3. The tick was removed with fine forceps and sent to the laboratory. Laboratory results confirmed that the tick belongs to the ixodes genus. Visual acquity was excellent in both eyes, intraocular pressures within normal limits and anterior and posterior segment examination unremarkable. After consultation with the microbiology department we decided not to prescribe any topical or systemic antibiotic as the evidence on prophylaxis after a tick bite is weak. Patient was informed in details about presenting symptoms and signs, both ocular and systemic, of tick bite related diseases and a letter was sent to her general practitioner. No further follow ups were made.

**Figure 3:**
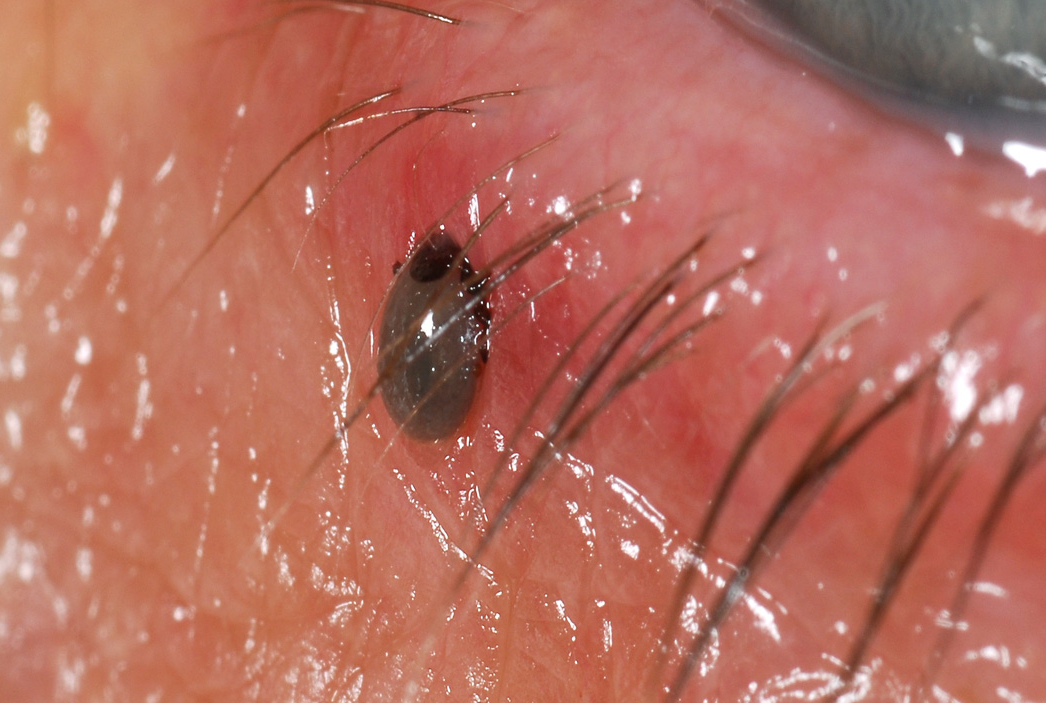
**lower lid tick infestation picture**.

## Conclusion

Tick Ixodes can be a vector of many diseases commonly Lyme disease which is caused by Borrelia burgdorferi. Ixodes is infected by Borrelia at larval stage when it feeds on infected mammals. Man is an occasional host. The transmission depends on several factors, especially on the duration of the tick's presence in the host body and on whether the tick is infected or not[1]. Ocular manifestations which are quite rare are described below. A nonspecific follicular conjunctivitis occurs in approximately 10% of patients with early Lyme disease. Keratitis occurs often within a few months of onset of disease and is characterized by nummular nonstaining opacities. Inflammatory syndromes, such as vitritis and uveitis, have been reported; in some cases, a vitreous tap is required for diagnosis. Neuro-ophthalmic manifestations include neuroretinitis, involvement of multiple cranial nerves, optic atrophy, and disc edema. Seventh nerve paresis can lead to neurotrophic keratitis. In endemic areas, Lyme disease may be responsible for approximately 25% of new-onset Bell's palsy[2]. Prevention measures include reducing the number of ticks with simple interventions and personal prevention measures, such as reducing the amount of exposed skin, use of tick repellents on exposed skin or clothing, and frequent tick checks to remove attached ticks expeditiously. However, none of these methods. has been demonstrated to decrease significantly the incidence of Lyme borreliosis in humans[3,4]. Only two strategies have been shown to do so. A recombinant outer surface protein A (OspA) vaccine was approximately 80% effective in clinical trials in the United States, and a single 200 mg dose of doxycycline given within 72 hours of an I. scapularis tick bite, was shown to be 87% effective[5].

Written informed consent was obtained from the patient for publication of this case report and accompanying images. A copy of the written consent is available for review by the Editor-in-Chief of this journal.

## Competing interests

The authors declare that they have no competing interests.

## Authors' contributions

VL has involved in writing this article, obtaining the consent form and pictures under the guidance of CG. All authors read and approved the final manuscript.
